# Correlation of Trace Mineral Status Between Cows and Their Calves: Insights from Paired Serum Samples

**DOI:** 10.3390/ani16060866

**Published:** 2026-03-10

**Authors:** Laura Fresco-Rey, Lucas Rigueira, Candela Fernández-Villa, Belén Larrán, Marta López-Alonso, Silvia Rojo-Montejo, Ramiro Fouz, Víctor Pereira, Marta Miranda

**Affiliations:** 1 Department of Anatomy, Animal Production and Clinical Veterinary Sciences, Faculty of Veterinary Medicine, Campus Terra, University of Santiago de Compostela, 27002 Lugo, Spain; laura.fresco.rey@rai.usc.es (L.F.-R.); lucas.rigueira@usc.es (L.R.); candela.fernandez.villa@usc.es (C.F.-V.); silvia.rojo@usc.es (S.R.-M.); ramiro.fouz@usc.es (R.F.); 2 Rof-Codina Veterinary Teaching Hospital, Faculty of Veterinary Medicine, Campus Terra, University of Santiago de Compostela, 27002 Lugo, Spain; 3 Department of Animal Pathology, Faculty of Veterinary Medicine, Campus Terra, University of Santiago de Compostela, 27002 Lugo, Spain; belen.larran.franco@usc.es (B.L.); marta.lopez.alonso@usc.es (M.L.-A.); victor.pereira@usc.es (V.P.)

**Keywords:** trace minerals, dairy cattle, calf, maternal–fetal bond, serum

## Abstract

This study assessed trace mineral levels (Co, Cr, Cu, Fe, Mn, Mo, Se, and Zn) in paired serum samples from multiparous Holstein Friesian cows and their calves after colostrum intake, to explore potential relationships between maternal and neonatal mineral status. The acid-digested samples were analyzed by inductively coupled plasma mass spectrometry. Serum levels of Co, Cu, Fe, and Se were significantly higher in cows than in calves (*p* < 0.001), while Zn levels were higher in calves. The levels of Cr, Mn, and Mo were similar in both groups. Overall, mineral deficiencies were more prevalent in cows, with Se being the most deficient element, followed by Zn, Cu, and Co. Calves were more deficient in Co and Mn than their mothers but were not generally deficient in Se. Serum levels of Cr, Cu, Mn, Mo, and Se were positively correlated in cows and their calves, suggesting that maternal mineral status influences neonatal mineral levels. Overall, these results provide insights into trace mineral dynamics in cow–calf pairs. Further studies are needed to clarify the relative contribution of placental and colostral mineral transfer.

## Simple Summary

The mineral levels of newborn calves could be influenced by the trace mineral status of their mothers. In this study we detected higher levels of minerals such as cobalt, copper, iron, and selenium in cows than in calves and higher levels of zinc in calves than in cows. Trace mineral deficiencies, particularly of selenium, were more common in cows than in calves. Importantly, cows with good mineral statuses were more likely to have calves with adequate mineral levels, confirming effective transfer of minerals before and after birth. These findings highlight the importance of correct mineral supplementation of cows during the dry period to ensure calves are born with the balanced mineral levels that are needed for a healthy start to life.

## 1. Introduction

Trace minerals, although required in very small amounts, play a crucial role in the health and productivity of dairy cattle. Elements such as cobalt (Co), chromium (Cr), copper (Cu), iron (Fe), iodine (I), manganese (Mn), molybdenum (Mo), selenium (Se), and zinc (Zn) are involved in essential physiological functions, including antioxidant defense, immune response and metabolic regulation [1,2].

Trace minerals are transferred from the dam to the calf, in a process that begins during gestation, through the placenta, and continues after birth, via colostrum [3,4,5]. During gestation, the fetus is entirely dependent on maternal nutrients, and the mineral status largely reflects that of the mother. Most minerals are efficiently accumulated in the fetal liver, although transfer of minerals such as I and Mn is less efficient [6,7,8]. Maternal mineral deficiencies or imbalances can therefore compromise fetal mineral reserves, potentially affecting neonatal health and growth [4,5]. After birth, colostrum serves as the primary source of trace minerals, vitamins, and immunoglobulins, supporting immune function, antioxidant protection, and the transition to extrauterine life [5,9,10,11]. Calves rely on colostrum for passive immunity and also to meet physiological challenges such as hypoglycemia, hypoxia, and oxidative stress, the latter of which arises from increased oxygen exposure and metabolic demands at birth [9,10,11,12,13,14]. The mineral content and quality of colostrum can be influenced by maternal factors such as parity, length of dry period, prepartum nutrition, and physiological stress, and supplementation with trace minerals during gestation can enhance both dam and calf health [15,16,17,18,19].

This research study provides a novel contribution to the study of cattle mineral status, as it is the first to evaluate trace mineral correlation by analyzing paired serum samples from dairy cows and their newborn calves in conventional intensive production systems. These systems are ideal for such studies because animals receive carefully formulated and mineral-supplemented diets, allowing controlled assessment of maternal–neonatal mineral dynamics. The aim of the study was to quantify levels of trace minerals (Co, Cr, Cu, Fe, Mn, Mo, Se, and Zn) in paired cow–calf samples after the calves had ingested colostrum to explore potential relationships between maternal and neonatal mineral status. These measurements enabled more precise characterization of mineral levels in cow–calf pairs, identification of potential deficiencies, and generation of information that could support optimized nutritional management and calf health during early life.

## 2. Materials and Methods

Animal sampling and handling procedures were approved by the Bioethics Committee of the Rof-Codina Clinical Veterinary Hospital (University of Santiago de Compostela, Spain), as detailed in the ‘Institutional Review Board Statement’ section.

### 2.1. Animals and Sampling

To carry out the present study, paired blood samples were taken from 52 multiparous (3rd and 4th lactation) Holstein Friesian cows and their 52 female calves after colostrum ingestion (16.4 ± 8.1 h after birth). Colostrum volume and quality were routinely assessed on the farm by recording the administered volume and measuring colostrum quality using a Brix refractometer. Only calves receiving 4–6 L of adequate-quality colostrum (Brix > 22%) and showing successful passive transfer (serum total protein > 5.8 g/dL at 24–48 h of life) [20] were included in the study. Calves were generally sampled 6–8 h after colostrum ingestion, although when cows calved during the night, sampling occurred later. During the 60-day dry period, cows were fed a total mixed ration (TMR), formulated to meet nutrient recommendations according to NASEM [21], consisting of 13 kg of corn silage, 7.25 kg of barley straw, 3.5 kg of rapeseed and 1 kg of a vitamin–mineral premix, with a DCAD of 0.43 mEq/100 g of dry matter (DM).

Blood samples were collected by coccygeal venipuncture (adult cows) and jugular vein venipuncture (calves), in 5 mL tubes with no additives (Vacuette^®^, Z Trace Elements Serum Clot Activator; Greiner bio-one, Kremsmünster, Austria). The samples were refrigerated (4–6 °C) and transported to the laboratory where they were centrifuged at 3000 rpm for 10 min to obtain the serum. Two aliquots of each sample were then obtained and frozen at −18 °C until further analysis.

### 2.2. Sample Preparation and Analysis

The serum samples were subjected to a simple acid digestion procedure, following the method described by Luna et al. [22]. Specifically, 1 mL of serum was mixed with 0.6 mL of concentrated nitric acid (69%) and 0.4 mL of hydrogen peroxide (33%) in propylene tubes. The mixture was maintained at 60 °C in open vessels for 12 h. The digested samples were diluted to 5 mL in Milli-Q water before being centrifuged at 2000 rpm for 5 min. The supernatant was then collected for immediate analysis. The ration (TMR) was oven-dried at 60 °C for 24 h, before being ground and sieved through a 0.5 mm mesh. Samples were subsequently acid digested using 8 mL of concentrated nitric acid (69%) and 2 mL of hydrogen peroxide (33%) in a microwave digestion system (Ethos Plus; Milestone, Sorisole, Italy).

The levels of Co, Cr, Cu, Fe, Mn, Mo, Se, and Zn in the prepared samples were determined by inductively coupled plasma–mass spectrometry (ICP-MS) (Agilent 7900 ICP-MS, Agilent Technologies, Tokyo, Japan). An internal Rh standard was continuously introduced online to correct instrumental drift and matrix-related effects. The analyses were carried out at the Research Infrastructures Unit of the USC (Lugo, Spain). This laboratory operates under a stringent analytical quality control protocol and holds ISO accreditation, ensuring the reliability and traceability of the results.

To guarantee the accuracy of the procedure, calibration curves (range 0.2 to 10,000 μg/L) were prepared daily with fresh standard solutions prior to analysis of plasma samples. The correlation coefficients for the detection responses of the ICP-MS instrument were higher than 0.999, and the relative standard deviations were below 5%. All samples were analyzed in triplicate. An analytical quality control procedure was implemented during the preparation and subsequent analysis of the samples. Analytical blanks were included during the processing of all batches, and the limit of detection (LOD) was calculated as 3 times the standard deviation of the blanks. The LOD values were low enough to allow determination of all elements. The accuracy of the method was verified using bovine serum samples spiked with appropriate concentrations of the elements (up to 2–10 times higher than the normal levels of samples). Overall, good recoveries were achieved for the spiked serum samples (ranging from 85.5–101.6%), and the accuracy of the determinations was therefore considered acceptable.

### 2.3. Statistical Analysis

The statistical analyses were conducted using SPSS for Windows (v.28.0). Data normality was assessed using the Kolmogorov–Smirnov test. Homogeneity of variances was assessed using Levene’s test prior to conducting ANOVA, and all models met the assumption of homoscedasticity. Differences between groups were examined using one-way ANOVA, and the correlations between the trace minerals were assessed using Pearson’s correlation coefficients. A significance level of *p* < 0.05 was applied in the tests.

## 3. Results and Discussion

**Figure 1:**
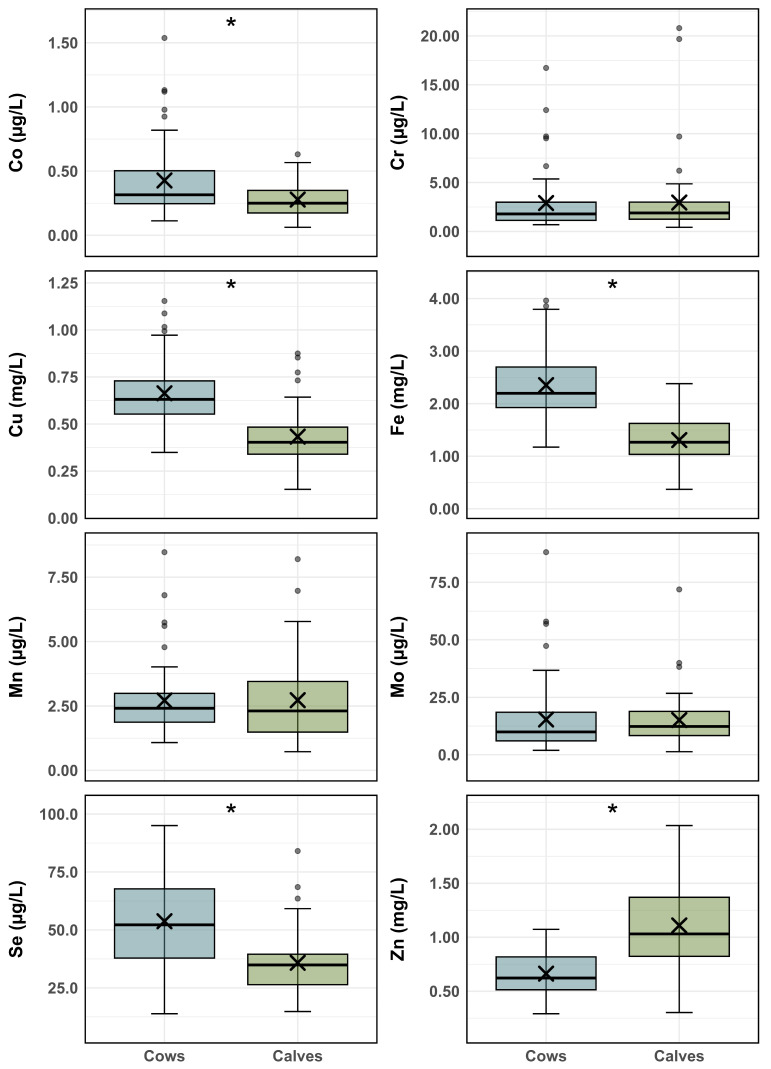
Box-and-whisker plot showing the serum concentrations of cobalt (Co), chromium (Cr), copper (Cu), iron (Fe), manganese (Mn), molybdenum (Mo), selenium (Se), and zinc (Zn) in cows and calves. * indicates a statistically significant difference *p* < 0.001. Boxes represent the interquartile range (25th–75th percentiles), with the horizontal line indicating the median and the cross (X) the mean. Whiskers extend to 1.5 × IQR, and circles indicate outliers.

The serum trace mineral concentrations of the cows and their calves are represented in Figure 1. Serum concentrations of Co, Cu, Fe, and Se were significantly higher (*p* < 0.001) in cows than in calves, whereas the Zn concentrations were significantly higher in calves than in their mothers (*p* < 0.001). Chromium, Mn, and Mo concentrations were very similar in cows and calves (*p* > 0.9) (Figure 1). A similar pattern was observed in a recent study in beef cattle, with higher serum Zn concentrations in calves than in their mothers, while the concentrations of other trace minerals were lower in calves than in the cows [18].

Mean serum trace mineral concentrations in calves were within the established reference ranges [23]. By contrast, the mean Se concentrations in cows (53.8 μg/L) were below the adequate range (65–140 μg/L) [23] (Figure 1). Indeed, Se was the most deficient trace mineral in adult cows (73.1%), followed by Zn (44.2% below 0.6 mg/L), Cu (36.5% below 0.6 mg/L), and Co (13.5% below 0.17 μg/L). Mineral deficiencies were less frequent in calves. Cobalt was the most deficient element (26.9% below 0.18 μg/L). Additionally, in 11.5% of calves, the Cu, Mn, and Zn concentrations were below the respective reference ranges—0.3 mg/L, 1 μg/L, and 0.6 mg/L [23]. Only 5.5% of calves had Se concentrations below 20 μg/L [23]. Interestingly, Co and Mn deficiencies were more frequent in calves than in cows, and none of the cows had Mn concentrations below the normal range (0.9–6 μg/L). Conversely, Se deficiency was the most frequent trace mineral deficiency in cows and the least frequent in calves. Iron concentrations were within the normal range in both groups. Chromium concentrations in adult cows were also within the normal range [24]; however, reference values for neonates are not available. In contrast to the previous elements, Mo concentrations exceeded the upper limit of the adequate range in 5.8% of calves (>15 μg/L) and 9.6% of cows (>35 μg/L).

Trace minerals are transferred from the mother via both the placenta and colostrum and accumulate in the fetal liver, which acts as a reservoir [5]. It has been reported that liver levels of Cu, Fe, Se, and Zn are higher in fetuses than in cows, while the levels of Co, Mn, and Mo are lower in fetuses [4]. The fetal hepatic reserve maintains the levels of trace minerals necessary for metabolic and enzymatic function, thus maintaining normal blood levels [2]. In the present study, deficiencies in trace minerals that accumulate in the liver (Cu, Fe, Se, and Zn) were less frequent in the calves than in their mothers. By contrast, deficiencies in trace minerals that accumulate in smaller quantities in the liver (Co and Mn) were more frequent in the calves. Therefore, although the concentrations of Se, Zn, and Cu were borderline in the mothers, the fetal mineral reserves were prioritized [5]. In fact, all of the calves had a generally good trace mineral status, and despite the deficient levels of Se in the mothers (73.1%), less than 6% of the calves had Se levels below the adequate range. In the present study, approximately 10% of the cows showed elevated Mo concentrations, which could theoretically reduce Cu absorption. Well-known examples in ruminants include the Cu–Mo–S interaction, whereby high concentrations of Mo and/or S lead to the formation of thiomolybdates that block Cu absorption, causing secondary Cu deficiency [2,25]. However, in serum, no negative association between serum Mo and Cu concentrations was observed (r = 0.466, *p* < 0.001), and no significant negative associations between Fe and other trace elements were detected.

Trace mineral concentrations (mg/kg DM) in the diet were as follows: Co 0.184, Cr 1.15, Cu 6.75, Fe 154, Mn 26.7, Mo 1.14, Se 0.115, and Zn 25.2. Trace mineral deficiencies are not common in intensive dairy systems, because the diets are formulated to meet the nutritional requirements at each stage of production [26,27]. However, during the dry period it is common for cows to receive rations not provided as a TMR, or that are unbalanced or include poorly formulated (or no) mineral supplementation. This often results in inadequate or inconsistent mineral intake and increases the likelihood of trace mineral deficiencies. Although the cows in the present study were fed a correctly mixed TMR providing 23.75 kg—unlike many herds, where cows in the dry period are not fed TMR—the diet still failed to meet NASEM requirements [21] for key trace minerals. Specifically, the dietary concentrations of Cu and Se were below recommended levels (Cu: 17, Se: 0.3), showing that even a balanced TMR can fall short if mineral supplementation is not appropriately adjusted.

When deficiencies do emerge, they are frequently linked to insufficient mineral inclusion and also to imbalances that promote antagonistic interactions among minerals. As previously noted, a Cu–Mo–S interaction, in which elevated Mo and/or S concentrations promote thiomolybdate formation and reduce Cu absorption, leads to secondary Cu deficiency [2,25]. Excess Fe can similarly interfere with the absorption of Co, Cu, Se, and Zn [2,28], while excess Ca may hinder Zn availability [2,25]. In the present case, antagonism does not appear to have occurred, as the dietary levels of Mo, Fe, and S (3.19 g/kg DM) are consistent with NASEM recommendations. Trace mineral status of dairy cattle primarily depends on dietary intake [2,27], and therefore, the deficiencies detected in the cows in the present study probably originated from insufficient supply during the dry period.

A recent study carried out by our research group revealed a high prevalence of trace mineral deficiency in Spain, even in conventionally managed dairy herds [26]. Moderate deficiency in trace minerals that play an important role in immune function and antioxidant defense, such as Cu, Mn, Se, and Zn, can compromise the immune system, increasing health risks for both cows and calves and causing significant economic losses in the sector [1,29,30].

**Figure 2:**
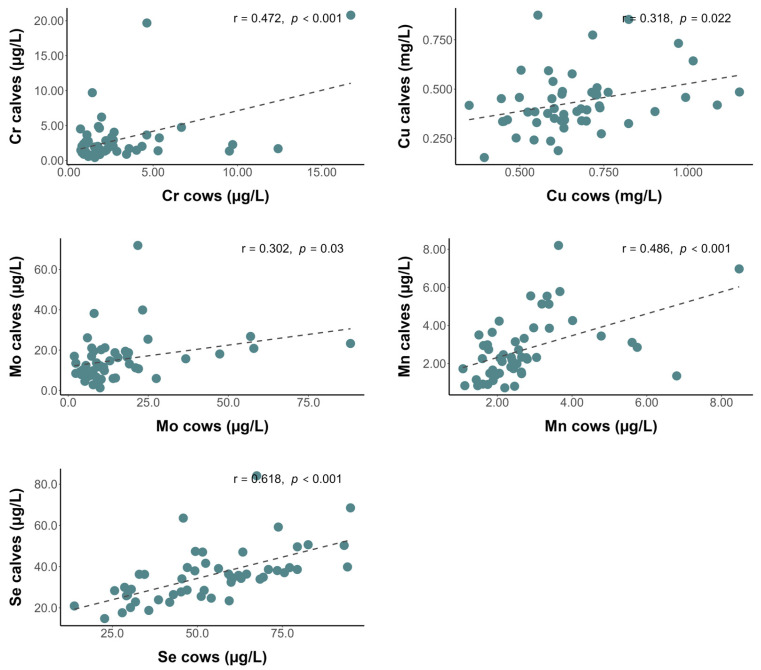
Pearson correlations plots for chromium (Cr), copper (Cu), manganese (Mn), molybdenum (Mo), and selenium (Se) concentrations in paired cow–calf serum samples.

The serum concentrations of Cr, Cu, Mn, Mo, and Se in the cows and calves were positively correlated (Figure 2), but those of Co, Fe, and Zn were not correlated. By contrast, Hurlbert et al. [18] observed a positive correlation in serum Co levels in beef cows and their calves but no such correlation for Cu, Mn, Mo, Se, or Zn. It is well established that the developing fetus depends on the supply of nutrients from the mother via the placenta and subsequently through colostrum; therefore, the trace mineral status reflects the maternal nutritional status [3,4,5,6,7,8,9]. Trace mineral transport across the placenta is not fully understood, and perinatal trace mineral transfer is complex and unclear [31]. However, studies based on mineral concentrations in livers of fetuses, conducted more than twenty years ago, demonstrated that most minerals (except iodine) are efficiently transferred from the mother to fetus [3,6,7,8].

Current studies have clearly demonstrated the permeability of trace minerals across the maternal–fetal interface. Calves born to mothers supplemented with trace minerals had higher concentrations of minerals in the blood, muscle, and liver [18]. Dávila Ruiz et al. [19] demonstrated that supplemented heifers had higher concentrations of Se in the placenta and tended to have greater vascularity in the placental cotyledon than control heifers, which ensured the successful transfer of minerals to the calf during gestation. Among the correlations between serum concentrations of trace minerals in cows and calves, Se concentrations in cows and calves were the most significant (r = 0.618; *p* < 0.001). It is well known that Se is one of the trace minerals with the greatest capacity to cross the placental barrier into fetal tissues. Selenium concentrations in the fetal liver are at least twice as high and positively correlated with maternal liver Se concentrations [25]. A recent study reported higher serum Se concentrations in female calves after birth (before ingestion of colostrum) than in male calves, suggesting that placental Se transfer or Se utilization in fetal tissues may be influenced by the calf’s sex or body weight [15]. In addition to placental transfer, colostrum is a very good source of Se [9], which could explain the strong correlation between Se levels in maternal and fetal blood; however, colostrum Se concentration was not measured in the present study.

In addition, positive correlations between Mn levels in maternal and fetal livers have been reported [25]. Similarly, Cu concentrations in whole blood were found to be positively correlated with copper concentrations in the liver [25,32]. A recent study observed a positive linear association between Cu levels in colostrum and serum in multiparous Jersey cows, while no correlations were observed for Fe or Zn [16]. Our findings are consistent with these previous studies, as we observed a positive correlation between serum Cu levels in the mother and calf. Therefore, the transfer of trace minerals from the mothers to their offspring is prioritized to ensure adequate blood trace minerals status and prevent deficiencies. The strongest correlation and the greatest transfer effect was observed for Se. In fact, despite the low concentrations of Se in the cows, the concentrations were below the adequate range in only 3 of the 52 calves.

This study has several limitations that should be considered when interpreting the results. First, it is a preliminary and exploratory investigation, and the relatively small sample size may restrict the generalizability of the findings. In addition, colostrum mineral concentrations were not measured, and therefore, we could not fully assess the potential pathways of trace mineral transfer through the placenta or colostrum, which limits the ability to quantify the relative contribution of each route to the neonatal status. Moreover, the herd under study was clearly deficient in some trace minerals, and the research should be replicated in herds with adequate trace mineral status to better distinguish physiological patterns from deficiency-related effects. Despite these limitations, this study provides relevant information, as paired cow–calf serum sampling is uncommon and allows a direct evaluation of maternal–neonatal trace mineral relationships. Overall, these limitations highlight the need for further research to better understand mineral transfer processes and to optimize supplementation strategies during the dry period.

## 4. Conclusions

Although serum concentrations of most trace minerals, except for Zn, were higher in cows than calves, the cows also showed a markedly higher prevalence of trace mineral deficiencies. It is possible that the results may have been influenced by the fact that the cows were not supplemented to meet recommended levels. Although the mineral status of the calves was generally adequate, definitive conclusions about neonatal adequacy cannot be drawn, given the lack of pre-colostrum sampling and the absence of colostrum mineral analysis. The strong positive correlations between maternal and calf mineral concentrations—particularly for Se—highlight the close relationship between cow and calf mineral status; based on serum measurements alone, maternal mineral balance is associated with neonatal mineral status at birth. Overall, these findings provide valuable insights into trace mineral dynamics in cow–calf pairs and emphasize the relevance of monitoring maternal mineral status for neonatal health. Further research is warranted to elucidate the contribution of mineral transfer via the placenta and colostrum, especially in herds with optimal mineral status, in order to provide more definitive guidance for supplementation strategies and neonatal health outcomes.

## Data Availability

The data that support the study findings are available from the corresponding author upon request.
